# Endurance Training Intensity Does Not Mediate Interference to Maximal Lower-Body Strength Gain during Short-Term Concurrent Training

**DOI:** 10.3389/fphys.2016.00487

**Published:** 2016-11-03

**Authors:** Jackson J. Fyfe, Jonathan D. Bartlett, Erik D. Hanson, Nigel K. Stepto, David J. Bishop

**Affiliations:** ^1^Institute of Sport, Exercise and Active Living, College of Sport and Exercise Science, Victoria UniversityMelbourne, VIC, Australia; ^2^College of Sport and Exercise Science, Victoria UniversityMelbourne, VIC, Australia; ^3^Western Bulldogs Football ClubMelbourne, VIC, Australia; ^4^Department of Exercise and Sport Science, University of North Carolina at Chapel HillChapel Hill, NC, USA

**Keywords:** concurrent training, interference, interval, continuous, training, intensity

## Abstract

We determined the effect of concurrent training incorporating either high-intensity interval training (HIT) or moderate-intensity continuous training (MICT) on maximal strength, counter-movement jump (CMJ) performance, and body composition adaptations, compared with single-mode resistance training (RT). Twenty-three recreationally-active males (mean ± SD: age, 29.6 ± 5.5 y; $\dot{\text{V}}{\text{O}}_{2\text{peak}}$, 44 ± 11 mL kg^−1^·min^−1^) underwent 8 weeks (3 sessions·wk^−1^) of either: (1) HIT combined with RT (HIT+RT group, *n* = 8), (2) work-matched MICT combined with RT (MICT+RT group, *n* = 7), or (3) RT performed alone (RT group, *n* = 8). Measures of aerobic capacity, maximal (1-RM) strength, CMJ performance and body composition (DXA) were obtained before (PRE), mid-way (MID), and after (POST) training. Maximal (one-repetition maximum [1-RM]) leg press strength was improved from PRE to POST for RT (mean change ± 90% confidence interval; 38.5 ± 8.5%; effect size [ES] ± 90% confidence interval; 1.26 ± 0.24; *P* < 0.001), HIT+RT (28.7 ± 5.3%; ES, 1.17 ± 0.19; *P* < 0.001), and MICT+RT (27.5 ± 4.6%, ES, 0.81 ± 0.12; *P* < 0.001); however, the magnitude of this change was greater for RT vs. both HIT+RT (7.4 ± 8.7%; ES, 0.40 ± 0.40) and MICT+RT (8.2 ± 9.9%; ES, 0.60 ± 0.45). There were no substantial between-group differences in 1-RM bench press strength gain. RT induced greater changes in peak CMJ force vs. HIT+RT (6.8 ± 4.5%; ES, 0.41 ± 0.28) and MICT+RT (9.9 ± 11.2%; ES, 0.54 ± 0.65), and greater improvements in maximal CMJ rate of force development (RFD) vs. HIT+RT (24.1 ± 26.1%; ES, 0.72 ± 0.88). Lower-body lean mass was similarly increased for RT (4.1 ± 2.0%; ES; 0.33 ± 0.16; *P* = 0.023) and MICT+RT (3.6 ± 2.4%; ES; 0.45 ± 0.30; *P* = 0.052); however, this change was attenuated for HIT+RT (1.8 ± 1.6%; ES; 0.13 ± 0.12; *P* = 0.069). We conclude that concurrent training incorporating either HIT or work-matched MICT similarly attenuates improvements in maximal lower-body strength and indices of CMJ performance compared with RT performed alone. This suggests endurance training intensity is not a critical mediator of interference to maximal strength gain during short-term concurrent training.

## Introduction

Simultaneously incorporating both endurance and resistance training (RT) into a periodised exercise program is termed concurrent training. Compared with RT alone, concurrent training has been reported to attenuate training-induced improvements in maximal strength, power, and skeletal muscle hypertrophy in most (Hickson, 1980; Craig et al., 1991; Hennessy and Watson, 1994; Kraemer et al., 1995; Bell et al., 2000), but not all (McCarthy et al., 2002; Balabinis et al., 2003), studies. The equivocal nature of this interference effect can possibly be attributed to between-study variations in the prescription of individual training variables, which may modulate the degree of interference seen with concurrent training (Fyfe et al., 2014).

Two training variables likely to be important in mediating the interference effect are endurance training intensity and/or volume (Wilson et al., 2012; Fyfe et al., 2014). Endurance training intensity is a particularly relevant practical consideration, given that high-intensity interval training (HIT) can be more effective for enhancing aerobic capacity (Milanovic et al., 2015), and also reducing cardiometabolic risk factors (Wisløff et al., 2007; Tjønna et al., 2008), compared with traditional moderate-intensity continuous training (MICT). Evidence also suggests that HIT protocols involving brief work intervals (~2–4 min) interspersed with periods of active or passive recovery (~1–3 min) are perceived as more enjoyable compared with MICT (Bartlett et al., 2011), and are well-tolerated in clinical populations (Wisløff et al., 2007; Tjønna et al., 2008). Thus, HIT represents an attractive exercise strategy for both athletic and clinical populations, with promising implications for exercise adherence.

Despite the efficacy of HIT for promoting positive health and performance outcomes (Wisløff et al., 2007; Tjønna et al., 2008; Milanovic et al., 2015), there is currently limited information on the effects of incorporating HIT compared with MICT into concurrent training programs. Indeed, studies independently examining the potential role of endurance training intensity upon interference during concurrent training are scarce (Silva et al., 2012). One study (Silva et al., 2012) simultaneously investigated the effects of endurance training intensity (i.e., continuous vs. interval training) and modality (i.e., cycling vs. running) on neuromuscular adaptations to 11 weeks of concurrent training in physically-active females. No differences for improvements in one-repetition maximum (1-RM) leg press strength were found between training groups performing either RT only (52.6%) or concurrent training incorporating either continuous cycling (39.1%), continuous running (41.1%), or interval running (46.8%). However, the endurance training protocols used were only matched for total exercise duration, and not total work, making it difficult to deduce the potential influence of training intensity in mediating any effect on training-induced maximal strength outcomes (Silva et al., 2012). Further work is therefore required to delineate the potential roles of endurance training intensity on interference to maximal strength, power and hypertrophy outcomes during concurrent training.

Concurrent endurance training may interfere with RT adaptations by either (i) compromising subsequent RT performance via exacerbating residual fatigue and/or substrate depletion, or (ii) attenuating post-exercise anabolic responses that govern increases in rates of muscle protein synthesis and subsequent muscle fiber hypertrophy (Fyfe et al., 2014). A single bout of high-intensity endurance exercise reduces force generating capacity of the exercised musculature for at least 6 h post-exercise (Bentley et al., 2000), with lower-intensity training reported to elicit less residual fatigue (Leveritt et al., 2000; de Souza et al., 2007). Prior endurance exercise also compromises subsequent RT performance by reducing maximal strength or limiting RT volume (de Souza et al., 2007; Tan et al., 2014), an effect exacerbated after higher-intensity interval compared with lower-intensity continuous endurance exercise (de Souza et al., 2007). Higher exercise intensities are also associated with further increases in the activity of kinases purported to limit muscle protein synthesis, including AMPK (5′ adenosine monophosphate-activated protein kinase) (Rose et al., 2009). Whether these factors render HIT a suitable endurance training strategy to employ during concurrent training, compared with MICT, with respect to modulating interference to RT adaptations, is therefore unclear.

Given the popularity and efficacy of HIT for improving aerobic capacity and metabolic health markers, the aim of this study was to determine the effect of 8 weeks of concurrent training incorporating either HIT or more traditional MICT on maximal strength, counter-movement jump (CMJ) performance, and body composition adaptations, compared with single-mode RT, in recreationally-active males. It was hypothesized that, compared with RT performed alone, (i) concurrent training incorporating either HIT or MICT would attenuate increases in maximal strength, CMJ performance, and lean mass, and (ii), this interference effect would be exacerbated when RT was combined with HIT, compared to with MICT. Identification of training variables that are critical mediators of the interference effect will allow for targeted exercise prescription to minimize interference during concurrent training.

## Methodology

### Participants

**Figure 1:**
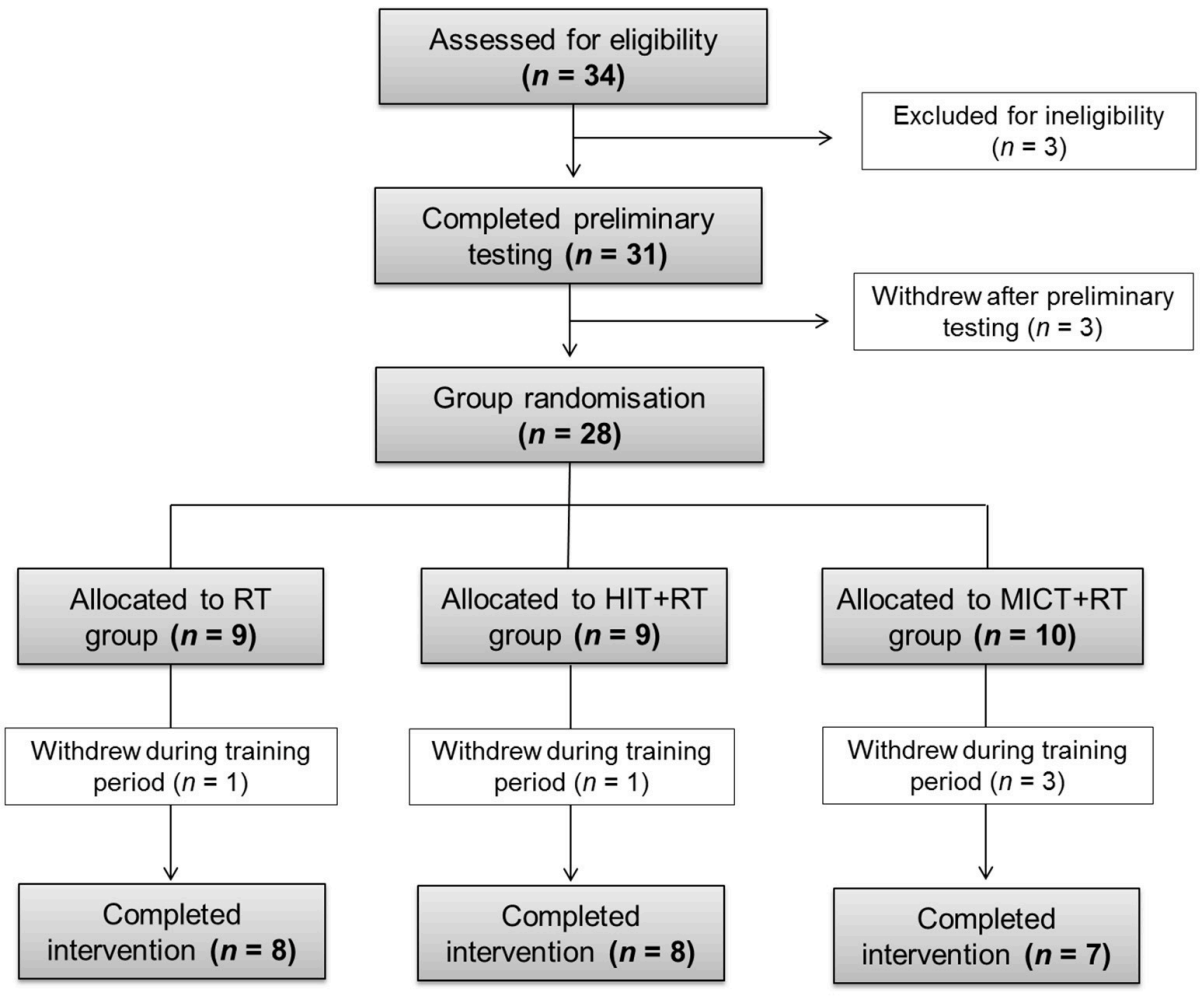
**Flow chart of participant progress through initial screening, preliminary testing, group randomization and final sample size for each training group**. HIT, high-intensity interval training; MICT, moderate-intensity continuous training; RT, resistance training.

Twenty-three recreationally-active males (mean ± SD: age, 29.6 ± 5.5 y; height, 182.4 ± 5.9 cm; body mass, 84.9 ± 11.4 kg) completed this investigation (see **Table 3** for baseline characteristics for each training group). A flow chart of the progression of participants through initial participant screening, group randomization, and to the final sample size included for each training group is shown in Figure 1. Participants were undertaking recreational exercise involving aerobic and/or resistance exercise at least twice per week for >30 min, and were free from any current cardiovascular abnormalities or musculoskeletal injuries to the upper or lower extremity. After being fully informed of study procedures and screening for possible exclusion criteria, participants provided written informed consent. All procedures were approved by the Victoria University Human Research Ethics Committee.

### Study overview

**Figure 2:**
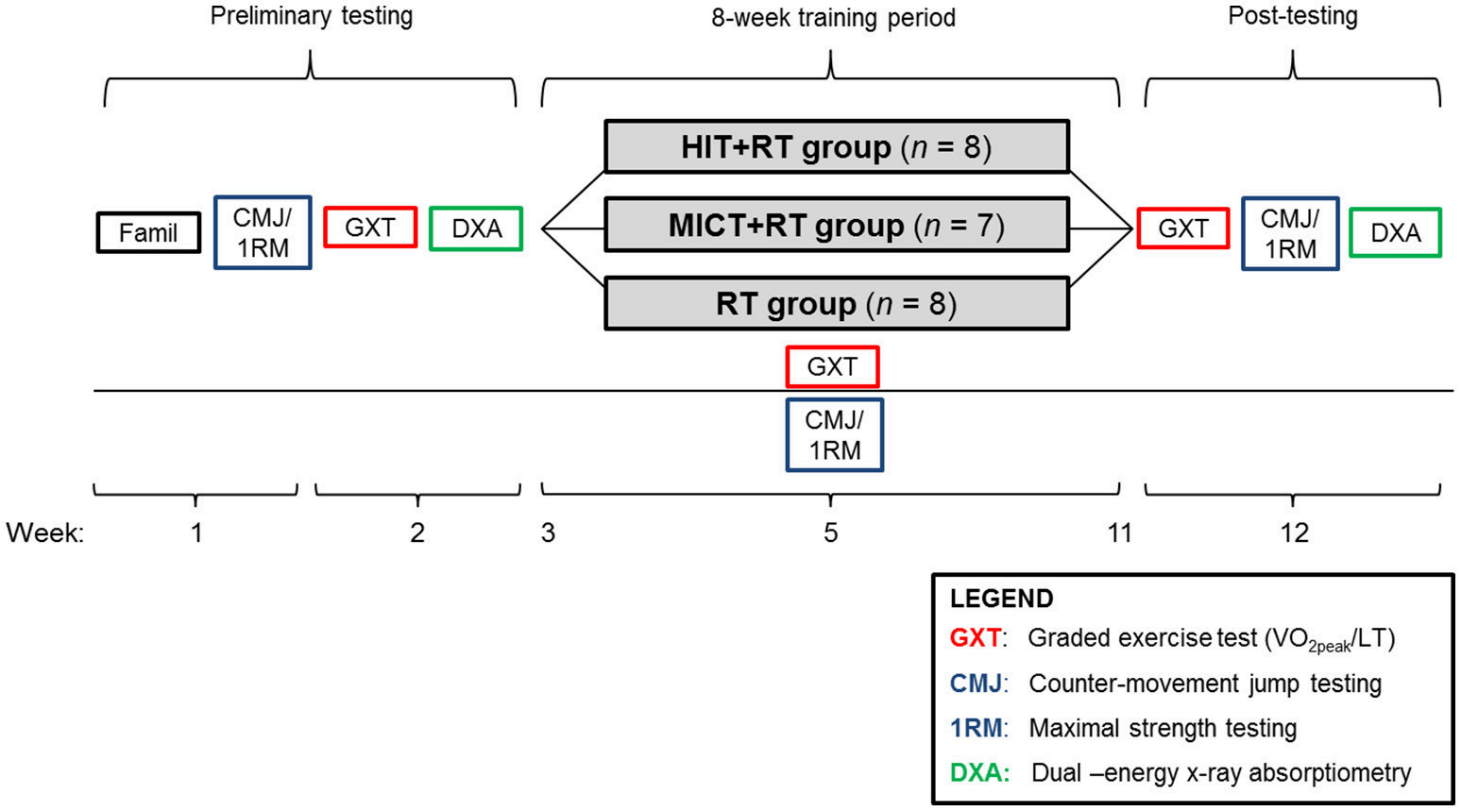
**Experimental overview**. HIT, high-intensity interval training; MICT, moderate-intensity continuous training; RT, resistance training; LT, lactate threshold; GXT, graded exercise test; CMJ, counter-movement jump; 1-RM, one-repetition maximum; Famil, familiarization; DXA, dual-energy x-ray absorptiometry.

The study followed a repeated-measures, parallel-group design. After preliminary testing, participants were ranked by baseline 1-RM leg press strength and randomly allocated to one of three training groups. Training groups consisted of (1) HIT cycling combined with RT (HIT+RT group, *n* = 8), (2) work-matched MICT cycling combined with RT (MICT+RT group, *n* = 7), and (3) RT performed alone (RT group, *n* = 8). Measures of aerobic capacity, maximal strength, and CMJ performance were obtained before (PRE), mid-way through (MID), and after completion (POST) of the training intervention (Figure 2). Body composition analysis (DXA) was performed only at PRE and POST. At least 72 h after preliminary testing, participants commenced 8 weeks of group-specific training performed three times per week. After training, the first post-training test [i.e., the graded exercise test (GXT) performed at POST] was undertaken at least 72 h after the final training session.

### Preliminary testing

#### Familiarization

Approximately 3–4 days before beginning preliminary testing, participants were familiarized with the CMJ, one-repetition maximum (1-RM) strength test and graded exercise test (GXT) protocols (each described subsequently).

#### Diet and exercise control

For 24 h prior to the GXT, CMJ/1-RM testing, and DXA, participants refrained from any structured exercise and recorded a detailed food diary. Participants were then asked to replicate this dietary intake as accurately as possible for the 24 h prior to each respective post-training test. On the morning of all testing sessions, participants reported to the laboratory after an ~8–10 h overnight fast. Prior to commencement of the training intervention, participants were asked to record a detailed 72 h food diary for the purposes of calculating average daily habitual energy and macronutrient intake. Dietary recalls were analyzed using Foodworks software (Version 6.0, Xyris Software, Australia). During the intervention period, participants were asked to maintain habitual dietary practices as closely as possible.

#### Graded exercise test (GXT)

The lactate threshold (LT) and peak aerobic power (W_peak_) were obtained during a GXT performed to volitional exhaustion on an electromagnetically-braked cycle ergometer (Lode Excalibur Sport, Groningen, The Netherlands). Prior to the GXT, a venous catheter was inserted into an antecubital forearm vein for subsequent blood sampling. The GXT consisted of 4-min work stages interspersed with 30 s of passive recovery. Participants maintained a pedaling cadence of 70 rpm during each work stage. The initial workload was set at 60, 90, or 120 W (to limit the number of stages to a maximum of 10, as determined during familiarization), and increased by 30 W for each subsequent stage until volitional exhaustion, defined as an inability to maintain a cadence >60 rpm. Venous blood samples (~1 mL) were drawn from the cannula at rest, and immediately following completion of each work stage. Whole-blood samples were immediately analyzed in duplicate for lactate concentration using an automated analyser (YSI 2300 STAT PLUS, Yellow Springs, OH). The lactate threshold was defined as the first workload that elicited a >1 mM increase in venous blood lactate concentration from baseline (Coyle et al., 1983) and was calculated using Lactate-OR software (ORRECO, Sligo, Ireland) (Newell et al., 2007). The W_peak_ was determined as previously described (Hawley and Noakes, 1992).

#### Peak oxygen uptake ($\dot{\text{V}}{\text{O}}_{2\text{peak}}$) test

Immediately following the GXT, a 5-min active recovery was initiated at 20 W, after which participants again cycled to volitional exhaustion at a workload corresponding to 105% of the W_peak_ achieved during the GXT. Participants were instructed to accelerate to a cadence of 90–100 rpm upon a 5-s countdown, and the test terminated when a cadence >60 rpm was no longer possible. Expired gases were sampled every 15 s during this test component using automated gas analysers (Moxus Modular $\dot{\text{V}}$O_2_ System, AEI Technologies, Pittsburgh, PA). A similar protocol has previously been reported to elicit $\dot{\text{V}}{\text{O}}_{2\text{peak}}$ values no different to that determined during a ramp incremental test performed 5 min previously (Rossiter et al., 2006). The gas analysers and pneumotach were calibrated prior to each test using known gas concentrations (21.0% O_2_ and 0.04% CO_2_, 16.0% O_2_ and 4.0% CO_2_) and a 3-L calibration syringe, respectively. The individual $\dot{\text{V}}{\text{O}}_{2\text{peak}}$ was defined as the highest two consecutive 15-s values achieved during the test. The test-retest reliability of GXT variables has been previously determined during repeated testing in our laboratory and yielded the following typical error values (expressed as a coefficient of variation [CV] ± 90% confidence intervals): LT (6.8 ± 1.2%), W_peak_ (5.5 ± 1.2%), and $\dot{\text{V}}{\text{O}}_{2\text{peak}}$ (6.5 ± 1.2%).

#### Maximal strength (1-RM) testing

Maximal strength was determined during a series of one-repetition maximum (1-RM) leg press and bench press attempts using a plate-loaded 45° incline leg press (Hammer Strength Linear, Schiller Park, IL) and standard bench press, respectively. After a standardized warm-up (5 and 3 repetitions at 50 and 70% estimated 1-RM, respectively), single repetitions of increasing load were attempted until the maximal load possible for one repetition was determined. Three minutes of recovery was allowed between 1-RM attempts. For the leg press, each repetition began in full knee extension with the heel placed at the bottom edge of the foot plate, and with a range of motion of 90° knee flexion/extension. Bench press repetitions were initiated from a position of full elbow extension, after which the barbell was lowered to the position of the chest and again lifted to full elbow extension. The test-retest reliability of 1-RM testing using similar protocols as the present study has been reported previously, with typical error values (expressed as a coefficient of variation [CV]) of 3.3% (Levinger et al., 2009) and 2.8% (McGuigan and Winchester, 2008) for 1-RM leg press and bench press, respectively.

#### Counter-movement jump (CMJ) testing

CMJ performance was assessed using a force plate (Fitness Technology, Skye, SA) interfaced with a linear position transducer (Ballistic Measurement System, Fitness Technology, Skye SA). After a standardized warm-up protocol (three submaximal unloaded CMJs), participants performed three maximal unloaded CMJs on the force plate with one min of passive recovery between each effort. The best of three trials were chosen for analysis. Jumps were initiated from a standing starting position, with the hands placed on the hips throughout the jump. Participants were instructed to self-select their jump depth and then accelerate as quickly as possible from the bottom position to achieve maximal concentric velocity and jump height. To allow for direct measurement of vertical displacement and movement velocity during each jump, the linear position transducer was attached to the center of mass of each participant via a weight belt. The test-retest reliability of CMJ variables was determined between the familiarization and preliminary testing sessions and yielded the following typical error values (expressed as a CV ± 90% confidence intervals): peak CMJ force (5.4 ± 1.5%), peak CMJ power (4.3 ± 1.5%), peak CMJ displacement (5.9 ± 1.5%), peak CMJ velocity (3.7 ± 1.5%), and maximal CMJ rate of force development (RFD) (19.3 ± 33.7%).

#### Body composition

Body composition was assessed via dual-energy x-ray absorptiometry (DXA; Discovery W, Hologic Inc., Bedford, MA) both pre- and post- training. DXA is a valid and reliable measurement tool for estimating total and regional body fat and lean mass (Nana et al., 2012). Typical error of measurement for regional lean mass has been reported as 1.3–1.7% (Nana et al., 2012) with strict control of diet and body position, while typical error for total lean and fat mass has been reported as 0.5 and 1.3%, respectively (Nana et al., 2012). To improve measurement reliability, participants were scanned in the fasted state and asked to refrain from exercise for 24 h before each scan. The scanner was calibrated daily, and the same certified densitometry technician performed and analyzed both the PRE and POST scans for each participant.

### Training intervention

Participants began the 8-week training intervention 3–5 days after completion of preliminary testing. All training groups performed an identical RT program on non-consecutive days (typically Monday, Wednesday, and Friday), with the HIT+RT and MICT+RT groups also completing the corresponding form of endurance exercise 10 min prior to commencing each RT session. Concurrent training was therefore always performed on the same day, with endurance exercise always preceding RT. All training programs were progressively modified to provide a sufficient overload stimulus, and are described in detail subsequently.

#### Endurance training

All cycling training sessions began with a 5-min warm-up performed at 75 W. The HIT protocol involved multiple 2-min intervals performed on an electromagnetically-braked cycle ergometer (Velotron RacerMate, Seattle, WA) at an intensity ranging between 120 and 150% of the LT, interspersed with 1 min of passive recovery. The MICT protocol involved continuous cycling performed on an electromagnetically-braked cycle ergometer (Velotron RacerMate, Seattle, WA) for a duration of between 15 and 33 min, and at a relative intensity ranging between 80 and 100% of the LT. All MICT sessions were work- and duration-matched to the corresponding HIT session (Edge et al., 2006). Progressive overload was applied by modulating the the number of intervals and relative exercise intensity (HIT) and the duration of cycling and relative exercise intensity (MICT) throughout the training program (Table 1). After re-testing of the GXT protocol at MID, relative endurance training intensities were adjusted as a percentage of the MID-training LT.

**Table 1:** **Progression of HIT and MICT prescription throughout the 8-week training intervention**.

		**HIT**		**MICT**	
**Week**	**Session**	**No. of 2-min intervals**	**Training intensity (% LT)**	**Duration of continuous training (min)**	**Training intensity (% LT)**
1	1	5	120	15	80
	2	6	120	18	80
	3	7	120	21	80
2	1	6	120	18	80
	2	8	120	24	80
	3	7	120	21	80
3	1	8	130	24	86.7
	2	9	130	27	86.7
	3	8	130	24	86.7
4	1	7	130	21	86.7
	2	6	130	18	86.7
	3	5	130	15	86.7
5	1	7	140	21	93.3
	2	8	140	24	93.3
	3	9	140	27	93.3
6	1	8	140	24	93.3
	2	9	140	27	93.3
	3	10	140	30	93.3
7	1	9	150	27	100
	2	11	150	33	100
	3	10	150	30	100
8	1	9	150	27	100
	2	7	150	21	100

*HIT, high-intensity interval training; MICT, moderate-intensity continous training; LT, lactate threshold*.

#### Resistance training (RT)

The RT program was performed three times per week on non-consecutive days. Sessions 1 and 3 of each training week included the leg press, bench press, seated row, leg extension and leg curl exercises. Session 2 of each training week included the leg press, flat dumbbell press, lat pulldown, dumbbell lunges and leg curl exercises. All exercises were performed at an intensity of between ~65 and 90% 1-RM (14- to 4-RM), with 2–3 min of recovery allowed between sets. For exercises where the 1-RM was not determined, load prescription was based on the maximum number of repetitions possible for a given load (i.e., the n-RM). For example, training prescription was set at 12 repetitions with a 14-RM load during the first week of training. During the first training session, loads were therefore adjusted until no more than 14 repetitions were possible with a given load for each exercise. During subsequent sessions, training loads were then increased concomitantly with changes in the n-RM prescription (Table 2). For each exercise, participants were instructed to perform the concentric portion of each repetition with a near-maximal to maximal intended movement velocity. The first three exercises of each session were preceded by a single warm-up set performed at approximately 75% of the planned workload for each respective exercise. Progressive overload was applied by altering the number of sets, repetitions, duration of rest periods, and relative exercise intensities throughout the training program (Table 2).

**Table 2:** **Progression of resistance training prescription throughout the 8-week training intervention**.

	**Week 1**	**Week 2**	**Week 3**	**Week 4**	**Week 5**	**Week 6**	**Week 7**	**Week 8**
**MON/FRI PROGRAM**								
Sets × repetitions	3 × 12	3 × 10	3 × 8	3 × 6	4 × 6	4 × 6	4 × 4	5 × 4
RM load	14	12	9	7	7	7	4	4
Rest period (min)	2	2	2	3	3	3	3	3
% 1-RM load	65	70	77.5	82.5	82.5	87.5	90	90
**WED PROGRAM**								
Sets × repetitions	3 × 12	3 × 12	3 × 10	3 × 10	3 × 8	3 × 8	4 × 6	3 × 6
RM load	14	14	12	12	9	9	7	7
Rest period (min)	2	2	2	2	2	2	2	2
% 1-RM load	65	65	70	70	77.5	77.5	87.5	87.5

*RM, repetition maximum; 1-RM, one-repetition maximum*.

### Physiological and psychological responses to exercise

To quantify the physiological and psycholigical responses to HIT and MICT, exercise heart rate (HR; Polar Electro, Kempele, Finland) and rating of perceived exertion (RPE; Borg's 6–20 scale) responses were collected at regular intervals during HIT and MICT sessions conducted in the first session of training weeks 1, 4, 5, and 8. For HIT sessions, HR and RPE data were collected after completion of each 2-min interval, while for MICT these data were collected at the equivalent time points during continuous exercise.

### Training load quantification

External training load (i.e., work performed) was matched for the HIT and MICT cycling protocols. Internal (i.e., perceived) training loads were also quantified during the intervention period using the session RPE (sRPE) method incorporating Borg's modified CR-10 scale (Foster et al., 2001). The sRPE method is a valid and reliable tool for quantifying internal training load for both endurance (Foster et al., 2001) and resistance exercise (Day et al., 2004). For the HIT+RT and MICT+RT groups, the sRPE for cycling was obtained 10 min following each cycling session (designated “cycling-only” internal training load), to determine internal training load for the HIT and MICT protocols. For all training groups, the sRPE was also obtained within 10 min after completion of RT as a marker of total-session training load. In addition to quantifying internal training load for all prescribed training, we also monitored the internal training load for exercise completed by participants outside of the study during the intervention period (i.e., non-prescribed training load) using a custom, web-based training diary. Participants were asked to record the sRPE, duration, and description of the activity within 30 min of completing each non-prescribed external training session. The non-prescribed training load was then added to the prescribed training load to determine the combined internal training load experienced by participants during the training intervention.

### Statistical analyses

The effect of training group on outcomes was evaluated via a two-way (time × group) analysis of variance with repeated-measures (RM-ANOVA) (SPSS, Version 21, IBM Corporation, New York, NY). Outcome variables were log-transformed before analysis to reduce non-uniformity of error (Hopkins et al., 2009). The magnitude of within- and between-group differences in outcomes was quantified using the standardized difference (effect size, ES) as previously described (Hopkins et al., 2009), with the default threshold of 0.2 defined as the smallest worthwhile effect. Magnitude-based inferences about effects were made by qualifying the effects with probabilities that reflected the uncertainty in the magnitude of the true effect (Batterham and Hopkins, 2005); 25–75%, possibly; 75–95%, likely; 95–99.5%, very likely; >99.5%, most likely. We considered substantial effects as those that were at least 75% “likely” to be greater than the smallest worthwhile effect [according to the overlap between the effect magnitude, the uncertainty in the magnitude of the true effect, and the smallest worthwhile effect (Batterham and Hopkins, 2005)]. A summary of all magnitude-based inference data for all within- and between-group comparisons for this study are presented in Supplementary Tables 1, 2, respectively. Exact *P*-values were also determined for each comparison, derived from paired (for within-group comparisons) or unpaired (for between-group comparisons) *t*-tests, with a Bonferroni correction applied to correct for multiple comparisons (SPSS, Version 21, IBM Corporation, New York, NY). Data are reported as the mean change (from PRE) ± 90% CL, unless otherwise specified.

## Results

### Training compliance

Training compliance (% of total sessions completed; mean ± SD) was as follows for each training group: HIT+RT, 98 ± 3%; MICT+RT, 97 ± 4%; RT, 98 ± 2%. There were no differences in training compliance between either RT vs. HIT+RT (mean difference ± 90% CL, 0.5 ± 2.5%; effect size [ES] ± 90% CL, 0.18 ± 0.84; *P* = 0.705), RT vs. MICT+RT (1.5 ± 3.3%; ES, 0.41 ± 0.90; *P* = 0.398), or HIT+RT vs. MICT+RT (0.9 ± 3.5%; ES, 0.21 ± 0.87; *P* = 0.635).

### Physiological and psychological responses to HIT and MICT

Average HR was higher during HIT compared with MICT during the first training session conducted in weeks 1, 4, and 5 (mean difference range ± 90% confidence interval, 13 ± 8 to 16 ± 10 beats min^−1^; ES range ± 90% confidence interval, 1.29 ± 0.85 to 1.45 ± 0.90; *P* ≤ 0.024). Similarly, average RPE was also higher for HIT compared with MICT during the first training session conducted in weeks 1, 4, 5, and 8 (2 ± 1 to 3 ± 2 AU; ES, 0.98 ± 0.86 to 1.49 ± 0.90; *P* ≤ 0.067).

### Internal training load

#### Weekly internal training load (cycling only)

**Figure 3:**
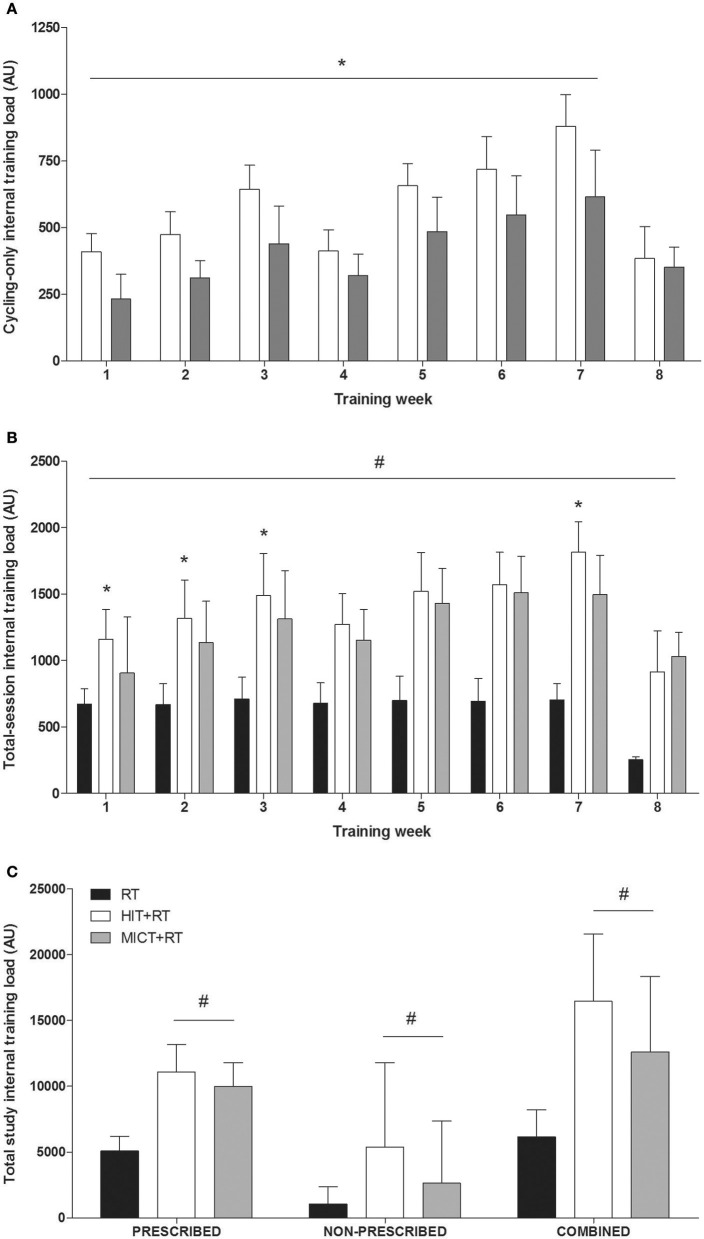
**Weekly cycling only (A) and total session (B) internal training load, and total prescribed, non-prescribed, and combined (prescribed + non-prescribed) internal training loads (C) during the 8-week training period for all training groups**. HIT, high-intensity interval training; MICT, moderate-intensity continous training; RT, resistance training. ^*^P < 0.05 vs. MICT; ^#^*P* < 0.05 vs. RT. Data shown are means ± SD.

Despite the HIT and MICT protocols being matched for external load (i.e., work performed), there were main effects of time (*P* < 0.001), group (*P* = 0.005), and a time × group interaction (*P* = 0.003) for cycling-only weekly internal training load (measured via sRPE). Cycling-only weekly internal training load (Figure 3A) was higher for HIT compared with MICT during training weeks 1–7 (% weekly difference range ± 90% confidence interval, 23 ± 15 to 49 ± 24%; ES range ± 90% confidence interval, 1.21 ± 0.87 to 2.07 ± 0.90; *P* ≤ 0.023).

#### Weekly internal training load (total session)

There were main effects of time (*P* < 0.001), group (*P* < 0.001), and a time × group interaction (*P* < 0.001), for total-session weekly internal training load. Total session weekly internal training load (Figure 3B) was higher during all training weeks for both HIT+RT (72 ± 30 to 244 ± 85%; ES, 2.77 ± 0.84 to 5.55 ± 0.89; *P* < 0.001) and MICT+RT (19 ± 34 to 302 ± 61%, ES, 0.32 ± 0.92 to 8.20 ± 0.89; *P* < 0.002) compared with RT. Total-session weekly internal training load was also higher for HIT+RT compared with MICT+RT at week 1 (31 ± 35%; ES, 0.70 ± 0.90; *P* < 0.001), week 2 (15 ± 22%; ES, 0.58 ± 0.90; *P* < 0.001), week 3 (13 ± 20%; ES, 0.54 ± 0.89; *P* = 0.007), and week 7 (19 ± 14%; ES, 1.09 ± 0.89; *P* = 0.004).

#### Total-study internal training loads

There were main effects of group for differences in total-study prescribed internal training load (*P* < 0.001) and total-study combined (i.e., prescribed + non-prescribed) internal training load (*P* = 0.001). Total-study prescribed internal training load (Figure 3C) was higher for both HIT+RT (119 ± 44%; ES, 3.26 ± 0.84; *P* < 0.001) and MICT+RT (108 ± 40%; ES, 3.28 ± 0.87; *P* < 0.001) compared with RT. There were also moderate effects for higher total study, non-prescribed, internal training load (Figure 3C) for HIT+RT compared with both RT (278 ± 624%; ES, 0.94 ± 0.92; *P* = 0.077) and MICT+RT (66.8 ± 49.9%; ES, 0.81 ± 0.87; *P* = 0.116). Total study combined (i.e., prescribed + non-prescribed) internal training load (Figure 3C) was higher for both HIT+RT (173 ± 72%; ES, 3.21 ± 0.84; *P* < 0.001) and MICT+RT (108 ± 70%; ES, 1.94 ± 0.87; *P* = 0.001) compared with RT. There was a moderate effect for a higher total-study, combined, internal training load for HIT+RT compared with MICT+RT (24 ± 25%; ES, 0.73 ± 0.88; *P* = 0.150).

### Habitual dietary intake

Baseline habitual dietary intake data are presented in Table 3. There was a main effect of group for differences in baseline average daily fat intake (*P* = 0.035). There were no substantial between-group differences in average daily protein intake at baseline (RT: 1.11 ± 0.37 g·kg^−1^·day^−1^; HIT+RT: 1.29 ± 0.34 g·kg^−1^·day^−1^; MICT+RT: 1.14 ± 0.28 g·kg^−1^·day^−1^; *P* > 0.05). A moderate effect for higher average total energy intake was noted for HIT+RT compared with RT (1079 ± 1369 kJ·day^−1^; ES, 0.66 ± 0.84; *P* = 0.208); this was due largely to a moderate effect for a higher fat intake for HIT+RT compared with both RT (23.4 ± 20.9 g·day^−1^; ES, 0.77 ± 0.84; *P* = 0.122) and MICT+RT (27.8 ± 19.9 g·day^−1^; ES, 1.02 ± 0.85; *P* = 0.057). There were also moderate effects for higher average daily carbohydrate intake for MICT+RT compared with both HIT+RT (26.3 ± 37.8 g·day^−1^; ES, 0.57 ± 0.89; *P* = 0.246) and RT (27.1 ± 38.9 g·day^−1^; ES, 0.58 ± 0.88; *P* = 0.245).

**Table 3:** **Physical characteristics and nutritional, exercise performance, and body composition data for all training groups**.

	**RT**			**HIT+RT**			**MICT+RT**		
	**PRE**	**MID**	**POST**	**PRE**	**MID**	**POST**	**PRE**	**MID**	**POST**
**PHYSICAL CHARACTERISTICS**									
Age (y)	28.6 ± 6.4	−	−	29.5 ± 2.1	−	−	30.8 ± 7.1	−	−
Height (cm)	182.7 ± 7.6	−	−	181.3 ± 5.8	−	−	183.3 ± 4.2	−	−
Body mass (kg)	86.6 ± 14	−	85.9 ± 14.6	82.6 ± 10.9	−	83.3 ± 11.7	85.5 ± 9.8	−	85.4 ± 8
**HABITUAL DIETARY INTAKE**									
Energy intake (kJ day^−1^)	7685 ± 1496	−	−	8764 ± 1596	−	−	7903 ± 1368	−	−
Carbohydrate intake (g day^−1^)	188.2 ± 32.3	−	−	189.0 ± 25.8	−	−	215.3 ± 48.1	−	−
Fat intake (g day^−1^)	78.8 ± 18.3	−	−	102.2 ± 27.7	−	−	74.4 ± 12.4	−	−
Protein intake (g day^−1^)	88.1 ± 18.9	−	−	94.4 ± 20.3	−	−	86.5 ± 12.7	−	−
**MAXIMAL STRENGTH**									
1-RM leg press (kg)	301 ± 59	350 ± 52^†^	412 ± 53^†^^#^^∧^	299 ± 56	341 ± 62^†^	383 ± 60^†^	291 ± 68	335 ± 69^†^	366 ± 60^†^
1-RM bench press (kg)	70 ± 21	80 ± 23^†^^#^^∧^	83 ± 22^†^	78 ± 15	85 ± 16^†^	90 ± 15^†^	79 ± 25	84 ± 22^†^	90 ± 24^†^
**CMJ VARIABLES**									
Peak force (N)	1847 ± 266	1887 ± 258^∧^	1977 ± 224^†^^#^^∧^	1777 ± 231	1779 ± 242	1784 ± 277	1872 ± 318	1814 ± 310^†^	1847 ± 232
Peak power (W)	2835 ± 272	2876 ± 199	3208 ± 468^†^	2699 ± 379	2804 ± 361	2799 ± 469	2917 ± 646	2949 ± 471	3065 ± 501
Peak velocity (m·s^−1^)	1.88 ± 0.41	1.90 ± 0.36	2.04 ± 0.34^†^	1.80 ± 0.18	1.82 ± 0.18	1.85 ± 0.19	1.83 ± 0.18	1.85 ± 0.13	1.94 ± 0.19^†^
Peak displacement (m)	0.48 ± 0.13	0.49 ± 0.12	0.52 ± 0.12	0.43 ± 0.06	0.45 ± 0.05	0.47 ± 0.07	0.48 ± 0.09	0.47 ± 0.05	0.51 ± 0.09
Maximal rate of force development (N s^−1^)	9730 ± 4532	9817 ± 3344	11709 ± 3305	9143 ± 4249	8164 ± 1060	8216 ± 1430	7641 ± 2052	9184 ± 6166	9281 ± 5500
**BODY COMPOSITION**									
Upper-body lean mass (kg)	39.7 ± 5.5	−	39.8 ± 5.1	38.6 ± 3.7	−	39.1 ± 3.5	39.3 ± 3.6	−	39.9 ± 2.9
Lower-body lean mass (kg)	21.2 ± 2.2	−	22.0 ± 1.8^†^	21.4 ± 2.5	−	21.9 ± 2.5	21.8 ± 1.5	−	22.5 ± 1.1
Total lean mass (kg)	60.9 ± 7.2	−	61.7 ± 6.5	60.1 ± 6.0	−	60.9 ± 5.5	61.0 ± 4.9	−	62.4 ± 3.7
Body fat (%)	18.2 ± 7.1	−	17.6 ± 6.9	17.0 ± 5.6	−	16.8 ± 5.7	18.8 ± 3.5	−	17.9 ± 3.4
**AEROBIC CAPACITY**									
$\dot{\text{V}}{\text{O}}_{2\text{peak}}$ (mL kg^−1^ · min^−1^)	42.2 ± 12.6	41.3 ± 8.9	40.7 ± 9.4	47.3 ± 13.4	47.4 ± 10.2	48.4 ± 10.0	43.4 ± 6.9	47.8 ± 9.7	45.4 ± 6.1
$\dot{\text{V}}{\text{O}}_{2\text{peak}}$ (L·min^−1^)	3.46 ± 0.78	3.49 ± 0.53	3.41 ± 0.64	3.80 ± 0.75	3.82 ± 0.52	3.96 ± 0.41	3.64 ± 0.38	4.10 ± 0.85^†^	3.86 ± 0.29
Lactate threshold (W)	145 ± 48	153 ± 48	155 ± 49	182 ± 53	182 ± 51	196 ± 47	159 ± 55	165 ± 40	174 ± 40
Peak aerobic power (W)	245 ± 56	243 ± 60	239 ± 56	279 ± 55	288 ± 57	301 ± 46^†^^‡^	267 ± 43	269 ± 47	279 ± 38

*Data shown are group means ± SD. RT, resistance training; HIT, high-intensity interval training; MICT, moderate-intensity continous training*.

† *P < 0.05 vs. PRE-training. Change from PRE substantially greater vs*.

# , HIT+RT;

∧ , MICT+RT;

‡ *, RT*.

### Maximal strength

#### 1-RM leg press strength

**Figure 4:**
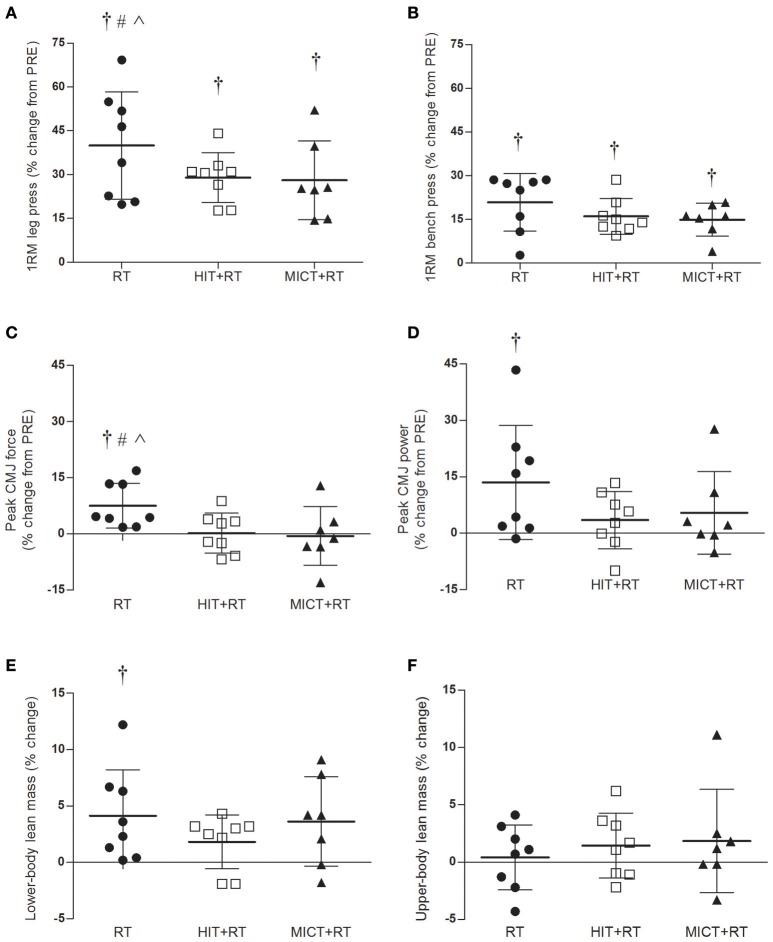
**Percentage changes in 1-RM leg press (A) and bench press (B) strength, peak counter-movement jump (CMJ) force (C) and power (D), and lower-body (E), and upper-body (F) lean mass between PRE- and POST-training**. RT, resistance training; HIT, high-intensity interval training; MICT, moderate-intensity continuous training; 1-RM, one-repetition maximum. Data shown are mean changes ± SD and individual participant responses. ^†^*P* < 0.05 vs. PRE-training. Change from PRE to POST substantially greater vs. ^#^HIT+RT, ^∧^MICT+RT.

There was a main effect of time for changes in 1-RM leg press strength (*P* < 0.001; Figure 4A), which was improved from PRE to POST for RT (mean difference ± 90% CL, 38.5 ± 8.5%; ES ± 90% CL, 1.26 ± 0.24; *P* < 0.001), HIT+RT (28.7 ± 5.3%; ES, 1.17 ± 0.19; *P* < 0.001) and MICT+RT (27.5 ± 4.6%, ES, 0.81 ± 0.12; *P* < 0.001). The change in 1-RM leg press strength from PRE to POST was greater for RT compared with HIT+RT (7.4 ± 8.7%; ES, 0.40 ± 0.40) and MICT+RT (8.2 ± 9.9%; ES, 0.60 ± 0.45), with trivial differences in this response between HIT+RT and MICT+RT (0.9 ± 8.1%; ES, 0.03 ± 0.30).

#### 1-RM bench press strength

There was a main effect of time for changes in 1-RM bench press strength (*P* < 0.001; Figure 4B), which was improved from PRE to POST for RT (20.5 ± 6.2%; ES; 0.50 ± 0.14; *P* < 0.001), HIT+RT (15.9 ± 2.6%; ES, 0.62 ± 0.09; *P* < 0.001), and MICT+RT (14.8 ± 2.3%; ES, 0.39 ± 0.06; *P* < 0.001). There were no substantial differences in the training-induced change in 1-RM bench press between RT and either HIT+RT (3.8 ± 6.1%; ES, 0.14 ± 0.22) or MICT+RT (4.7 ± 6.1%; ES, 0.15 ± 0.20).

### Counter-movement jump (CMJ) performance

There was a time × group interaction (*P* = 0.041) for changes in peak CMJ force, and main effects of time for changes in peak CMJ power (*P* = 0.008), peak CMJ velocity (*P* = 0.012), and peak CMJ displacement (*P* = 0.007).

#### Peak CMJ force

Peak CMJ force (Figure 4C) increased from PRE to POST for RT (7.4 ± 3.4%; ES, 0.46 ± 0.20; *P* = 0.008), with this change almost completely attenuated for both HIT+RT (0.1 ± 3.6%; ES, 0.00 ± 0.23; *P* = 0.979) and MICT+RT (−0.8 ± 4.9%; ES, −0.04 ± 0.26; *P* = 0.790). There were also moderate effects for a greater PRE to POST change in peak CMJ force for RT compared with both HIT+RT (6.8 ± 4.5%; ES, 0.41 ± 0.28) and MICT+RT (9.9 ± 11.2%; ES, 0.54 ± 0.65).

#### Peak CMJ power

Peak CMJ power (Figure 4D) increased from PRE to POST for RT (12.6 ± 10.5%; ES, 1.09 ± 0.85; *P* = 0.035), but not for either HIT+RT (3.2 ± 5.6%; ES, 0.20 ± 0.34; *P* = 0.266) or MICT+RT (5.0 ± 6.1%; ES, 0.19 ± 0.23; *P* = 0.241). The PRE to POST change in peak CMJ power was, however, not substantially different for RT compared with either HIT+RT (5.1 ± 7.3%; ES, 0.38 ± 0.56) or MICT+RT (3.5 ± 8.7%; ES, 0.21 ± 0.54).

#### Peak CMJ velocity

Peak CMJ velocity (Table 3) was increased from PRE to POST for RT (9.6 ± 8.2%; ES, 0.29 ± 0.24; *P* = 0.099) and MICT+RT (6.0 ± 4.0%; ES, 0.40 ± 0.26; *P* = 0.015), but not for HIT+RT (2.6 ± 4.8%; ES, 0.17 ± 0.31; *P* = 0.306). There were no substantial between-group differences in the PRE to POST change in peak CMJ velocity.

#### Peak CMJ displacement

Peak CMJ displacement (Table 3) was not substantially different between PRE and POST for either RT (9.5 ± 10.0%; ES, 0.22 ± 0.22; *P* = 0.108) or MICT+RT (7.0 ± 8.5%; ES, 0.34 ± 0.40 *P* = 0.129). However, there was a small effect for increased peak CMJ displacement for HIT+RT (7.8 ± 9.1%; ES, 0.50 ± 0.56; *P* = 0.134). There were no substantial between-group differences for the PRE to POST change in peak CMJ displacement.

#### Maximal CMJ rate of force development (RFD)

There was a moderate effect for increased maximal CMJ RFD between PRE and POST for RT (25.4 ± 26%; ES, 0.43 ± 0.39; *P* = 0.152), with no substantial change for either HIT+RT (−4.9 ± 11.8%; ES, −0.12 ± 0.29; *P* = 0.709) or MICT+RT (10.0 ± 33.5%; ES, 0.29 ± 0.91; *P* = 0.536). The PRE to POST change in maximal CMJ RFD was also substantially greater for RT compared with HIT+RT (24.1 ± 26.1%; ES, 0.72 ± 0.88).

### Body composition

There were main effects of time for changes in both lower-body lean mass (*P* < 0.001) and total lean mass (*P* = 0.006).

#### Lower-body lean mass

Lower-body lean mass (Figure 4E) similarly increased from PRE to POST for RT (4.1 ± 2.0%; ES; 0.33 ± 0.16; *P* = 0.023) and MICT+RT (3.6 ± 2.4%; ES; 0.45 ± 0.30; *P* = 0.052); however, this change was attenuated for HIT+RT (1.8 ± 1.6%; ES; 0.13 ± 0.12; *P* = 0.069). There were only trivial effects for between-group differences in the training-induced change in lower-body lean mass for RT compared with HIT+RT (2.2 ± 2.8%; ES, 0.18 ± 0.23) and for HIT+RT compared with MICT+RT (1.7 ± 3.1%; ES, 0.16 ± 0.28).

#### Upper-body lean mass

Changes in upper-body lean mass (Figure 4F) between PRE and POST were trivial in magnitude for RT (0.4 ± 1.9%; ES; 0.02 ± 0.19; *P* = 0.719), HIT+RT (1.4 ± 2.0%; ES; 0.13 ± 0.17; *P* = 0.198), and MICT+RT (1.8 ± 2.9%; ES; 0.17 ± 0.28; *P* = 0.325).

#### Total lean mass

Total lean mass (Table 3) was not substantially different from PRE to POST for RT (1.6 ± 1.4%; ES; 0.12 ± 0.10; *P* = 0.102) or MICT+RT (2.4 ± 2.4%; ES; 0.27 ± 0.26; *P* = 0.151). There was a statistically-significant, although trivial in magnitude, change in total lean mass for HIT+RT (1.6 ± 1.1%; ES; 0.14 ± 0.09; *P* = 0.038).

#### Body fat percentage

Body fat percentage (Table 3) was not substantially changed from PRE to POST for RT (−0.6 ± 1.0%; ES; −0.08 ± 0.17; *P* = 0.372), HIT+RT (−0.2 ± 0.9%; ES; −0.03 ± 0.15; *P* = 0.659), or MICT+RT (−0.9 ± 1.0%; ES; −0.25 ± 0.30; *P* = 0.115).

### Aerobic capacity

There was a main effect of time for changes in the lactate threshold (*P* = 0.005), and main effects for time (*P* = 0.036) and a time × group interaction (*P* = 0.041) for changes in peak aerobic power.

#### Peak oxygen consumption ($\dot{\text{V}}{\text{O}}_{2\text{peak}}$)

There were small effects for increased absolute peak oxygen consumption ($\dot{V}O$_2*peak*_; Table 3) between PRE and POST for both HIT+RT (5.3 ± 2.7%; ES, 0.25 ± 0.12; *P* = 0.162) and MICT+RT (6.1 ± 5.0%; ES, 0.27 ± 0.22; *P* = 0.103), with no change for RT (−0.6 ± 6.4%; ES, −0.02 ± 0.21; *P* = 0.876). There were no substantial differences in the PRE to POST change in $\dot{\text{V}}{\text{O}}_{2\text{peak}}$ between HIT+RT and MICT+RT.

#### Lactate threshold (LT)

Lactate threshold (LT; Table 3) was increased from PRE to POST for MICT+RT (12.6 ± 8.0%; ES, 0.30 ± 0.18; *P* = 0.107), but was not substantially different for either HIT+RT (8.3 ± 6.5%; ES, 0.20 ± 0.15 *P* = 0.054) or RT (7.4 ± 9.4%; ES, 0.13 ± 0.16; *P* = 0.080). There were no substantial between-group differences in the PRE to POST change in LT.

#### Peak aerobic power (W_peak_)

There were small and trivial effects, respectively, for increased peak aerobic power (W_peak_; Table 3) between PRE and POST for HIT+RT (8.8 ± 4.1%; ES, 0.31 ± 0.14; *P* = 0.010) and MICT+RT (4.9 ± 4.8%; ES, 0.19 ± 0.18; *P* = 0.096), with no change for RT (−2.2 ± 6.5%; ES, −0.06 ± 0.17; *P* = 0.515). The PRE to POST change in W_peak_ was also greater for HIT+RT compared with RT (11.3 ± 8.1%; ES, 0.35 ± 0.24), but not MICT+RT (7.3 ± 7.8%; ES, 0.24 ± 0.25).

## Discussion

This is the first investigation to compare the effects of HIT and work-matched MICT on adaptations to maximal strength, CMJ performance, and lean mass when performed concurrently with RT. The main findings of this study were that, compared with RT performed alone, concurrent training incorporating either HIT or work-matched MICT cycling similarly attenuated maximal lower-body strength development and improvements in peak CMJ force and power, while increases in lower-body lean mass were attenuated with concurrent training incorporating HIT, but not MICT.

Previous studies have observed attenuated maximal strength development following concurrent training incorporating HIT (Kraemer et al., 1995; Chtara et al., 2008), MICT (Craig et al., 1991; Gergley, 2009), or combinations of both (Hickson, 1980; Bell et al., 2000). However, it is unclear whether endurance training intensity might be important for mediating any interference effect to maximal strength development. The major finding of this study was that compared with performing RT alone both HIT and MICT attenuated maximal lower-body strength to a similar extent, but had no influence on upper-body strength development, when performed concurrently with RT. This was contrary to our hypothesis, as it was expected that interference to RT adaptations would be exacerbated in the HIT+RT group. Given that the HIT and MICT protocols employed in the present study were both duration- and work-matched, this observation lends support to the notion that endurance training volume (i.e., total work performed) might be a more critical mediator of interference to maximal strength gain during concurrent training than endurance training intensity (Wilson et al., 2012; Jones et al., 2013). Work by Jones et al. (2013) showed that altering the ratio of concurrent training, so that resistance- and endurance-like isokinetic contractions were performed at either a 1:1 or 3:1 weekly frequency ratio, led to compromised strength gain only when resistance and endurance exercise were both performed every session (i.e., with a 1:1 ratio). Moreover, performing maximal-intensity, low-volume, sprint interval cycling (i.e., a modified 20-s Wingate protocol) concurrently with RT does not interfere with maximal strength or lean mass improvements after 12 weeks of training (Cantrell et al., 2014). These observations, together with our present data, suggest that endurance training intensity may not be a critical mediator of interference to maximal strength gain with concurrent training, at least when total work is matched.

The observation of limited interference to maximal upper-body strength gain is in agreement with most (Hunter et al., 1987; Craig et al., 1991; Kraemer et al., 1995), but not all (Hennessy and Watson, 1994), concurrent training studies employing lower-body endurance training modalities; this suggests the mechanisms underlying this interference effect are local rather than systemic (Wilson et al., 2012). One mechanism by which concurrent endurance training may mediate any local interference effect is by compromising the quality of subsequent RT sessions (i.e., residual fatigue from prior endurance exercise) (Fyfe et al., 2014). Endurance exercise induces residual fatigue of the exercised musculature, which persists for at least 6 h post-exercise (Bentley et al., 2000), and is exacerbated after high-intensity interval vs. lower-intensity continuous endurance exercise (de Souza et al., 2007). However, whether the endurance training protocols employed in the present study elicited divergent effects on residual fatigue is unclear, although no negative effects of prior endurance exercise on planned RT intensities or volumes were observed for both the HIT+RT and MICT+RT groups. Another mechanism by which maximal strength may be compromised during concurrent training is via a concomitant attenuation in skeletal muscle hypertrophy, which may contribute to a reduction in force generating capacity. The observation of a similar attenuation to maximal lower-body strength gain in both concurrent training groups, together with the attenuated lean mass gain of the lower body for the HIT+RT group, suggests the interference to maximal strength gains may have been mediated by non-hypertrophic mechanisms. However, as no measures of training-induced changes in markers of muscle activation or neuromuscular fatigue were obtained, these mechanisms remain speculative.

Another aspect of adaptation to RT that may be attenuated during concurrent training is the ability to generate force rapidly (Kraemer et al., 1995; Häkkinen et al., 2003; Chtara et al., 2008), which is critical for power development. Adaptations to power development may be more susceptible to an interference effect during concurrent training compared with interference to maximal strength or hypertrophy (Häkkinen et al., 2003; Wilson et al., 2012). For example, 21 weeks of concurrent training attenuated improvements in isometric RFD compared with RT performed alone, with no detectable interference to 1-RM strength or maximal isometric force gains (Häkkinen et al., 2003). Moreover, a meta-analysis (Wilson et al., 2012) identified greater discrepancies between concurrent training and single-mode RT in effect sizes for lower-body power development (0.55 vs. 0.91, respectively) compared with differences in effect sizes for muscle hypertrophy (0.85 vs. 1.23, respectively) or maximal strength (1.44 vs. 1.76, respectively) development. We employed a CMJ protocol as a measure of explosive lower-body jumping performance. Jumping ability is considered an important element of successful athletic performance (Markovic, 2007), and indices of CMJ performance, including peak CMJ force and velocity, but not peak displacement, correlate with 20 and 30-m sprint times in youth soccer players (Chamari et al., 2004). Compromised improvement in either of these variables may therefore coincide with reduced performance during sport-specific activities such as acceleration and changing of direction.

In agreement with the interference to maximal lower-body strength development, concurrent training incorporating either HIT or MICT similarly attenuated improvements in peak CMJ force and power compared with RT performed alone. We also noted a substantially greater training-induced change in maximal CMJ rate of force development (RFD) for the RT group compared with the HIT+RT group. This same interference effect was, however, not observed with other CMJ variables, including peak velocity and displacement. Changes in peak CMJ velocity tended to be lower on average for the HIT+RT group; however, between-group differences for the change in peak CMJ velocity were trivial to small in magnitude (see Supplementary Table 2). Previous work by Chtara et al. (2008) found that performing HIT running concurrently with circuit-style RT attenuated improvements in several CMJ performance variables, including peak CMJ force, peak CMJ power, and jumping height. However, others have found no interference to vertical jump height improvements with concurrent training incorporating high-intensity running, compared with RT alone (Balabinis et al., 2003). Our data lend support to the notion that concurrent training interferes with RT-induced improvements in peak CMJ force and power, which appears to be primarily related to attenuated improvement in peak CMJ force rather than velocity. Moreover, the attenuation of peak CMJ force and power with concurrent training may be unrelated to the intensity of endurance training employed, at least when training is compared on a work-matched basis.

Despite our observations of interference to maximal strength gain and improvements in peak CMJ force, power, and RFD, there was little evidence this could be attributed to between-group differences in muscle mass gain. Previous studies have reported attenuated markers of muscle hypertrophy following concurrent training incorporating combinations of moderate- and high-intensity endurance training (Kraemer et al., 1995; Bell et al., 2000), compared with RT performed alone. However, others have observed no evidence of interference to muscle hypertrophy following lower-intensity, continuous endurance training (McCarthy et al., 2002; Lundberg et al., 2013). Whether the intensity of endurance training employed played a role in any interference to muscle hypertrophy development is therefore unclear. While similar increases in lower-body lean mass were noted for the MICT+RT group compared with RT performed alone, this improvement was attenuated for the HIT+RT group. Despite these differences, only trivial effects (ES, 0.18 and 0.16 for HIT+RT and MICT+RT compared with RT, respectively) were observed for between-group differences in the training-induced change in lower-body lean mass. Our data suggests that, on an external work-matched basis, performing higher-intensity endurance training concurrently with RT may compromise lean mass gain, which is specific to the musculature involved in both exercise modalities. Regardless, any small effect of concurrent training on lean mass responses were not reflected in the training-induced changes in both maximal strength and CMJ variables, suggesting that interference to these measures may be mediated by non-hypertrophic (and potentially neural) mechanisms.

It is possible the degree of RT-induced hypertrophy in the present study may have affected the likelihood of detecting clear between-group effects for interference to muscle hypertrophy with concurrent training. Whole-body lean mass gains observed in the present study (700–1400 g) are, however, similar to those reported in other studies utilizing DXA as a measure of lean mass gain (300–2300 g) following 6 (Candow et al., 2006) or 12 (Rakobowchuk et al., 2005) weeks of RT in the absence of targeted protein supplementation. Nevertheless, between-study differences in RT prescription may impact upon the degree of training-induced lean mass gain. The RT program in the present study was designed primarily to elicit improvements in maximal strength, with a linear progression from high-volume, moderate-intensity RT, to low-volume, high-intensity RT. While this increase in relative exercise intensity was likely favorable for maximizing strength gain, the reduced volumes associated with higher training intensities may have been suboptimal for maximizing skeletal muscle hypertrophy (Burd et al., 2010). In addition to training prescription, dietary protein supplementation may also further increase lean mass gain consequent to RT (Phillips and Van Loon, 2011). As the participants in the present study were not provided with protein supplementation, this may have also limited the degree of training-induced muscle hypertrophy, and should be a consideration for future studies. Indeed, the self-reported protein intakes of the participants in the present study (1.11–1.29 g·kg^−1^·day^−1^) may have been lower than optimal for promoting hypertrophy (1.3–1.8 g·kg^−1^·day^−1^) (Phillips and Van Loon, 2011). Nevertheless, given average daily protein intake was similar between training groups at baseline, and participants were asked to maintain habitual dietary practices during the intervention period, it is anticipated that between-group differences in training outcomes were not mediated by differences in amino acid availability.

In addition to quantifying internal training load for training sessions performed as part of the training intervention (i.e., prescribed training load), a custom, web-based training diary was used to also quantify internal training load for all training sessions participants completed outside of the study during the intervention period (i.e., non-prescribed training load). This was employed primarily as a surrogate measure training completed externally by participants, which may have influenced adaptation to our training intervention. As expected, total training load responses were substantially higher for both concurrent training groups compared with the RT group. Using this approach, it was also found that the non-prescribed internal training load was higher for the HIT+RT group compared with both the MICT+RT and RT groups, which contributed to a higher total study, combined internal training load for HIT+RT compared with MICT+RT. This suggests overall training volume may actually have been higher for the HIT+RT group, despite our HIT intervention being work-matched with MICT. Despite the between-group differences in internal training load responses, the precise relationship between internal training load and external work remains unclear as various factors (e.g., wellness markers such as perceived sleep quality and levels of muscle soreness, etc.) may modify the internal: external load relationship (Gallo et al., 2016; Saw et al., 2016) and therefore perceived training stress to a given training stimulus. Nevertheless, given the discrepancy in total internal training load between the HIT+RT and MICT+RT groups, it is difficult to deduce whether differences in outcomes such as lean mass changes are mediated by endurance training intensity or total training volume *per se*. Moreover, as lower-body 1-RM strength was similarly attenuated for the HIT+RT and MICT+RT groups compared with the RT group, this potentially suggests a superiority of HIT compared with MICT for promoting maximal strength gain during concurrent training, when compared on an internal training load-matched basis.

There is accumulating evidence for the greater efficacy of HIT for improving $\dot{\text{V}}{\text{O}}_{2\text{peak}}$ compared with MICT (Milanovic et al., 2015). However, it has also been shown that improvements in $\dot{\text{V}}{\text{O}}_{2\text{peak}}$ and the lactate threshold are similar after work-matched HIT and MICT (Edge et al., 2006). Our results suggest that, on a work-matched basis and when performed concurrently with RT, HIT, and MICT similarly increase $\dot{\text{V}}{\text{O}}_{2\text{peak}}$, the LT and W_peak_, although HIT was more effective in improving the W_peak_ compared with MICT. Improvements in these parameters were similar despite internal training load being substantially higher for HIT compared with MICT. These observations question the potency of HIT compared with traditional MICT for improving markers of aerobic capacity during concurrent training, although direct measures of endurance performance (e.g., distance- or work-based cycling time trial) were not evaluated. The present data also suggest that these divergent exercise intensities do not differentially modulate interference to maximal strength gain, at least on a work-matched basis, and after 8 weeks of training in recreationally-active males. It remains to be determined whether more prolonged periods of concurrent training, incorporating either HIT or MICT as the predominant endurance training modality, are associated with divergent effects on interference to RT adaptations.

It is clear there are a multitude of potential training variables associated with concurrent training (e.g., endurance and RT volume, intensity, and modality, training frequency, order of resistance and endurance training and between-mode recovery), each of which may play a role in mediating the interference effect (further discussed in Fyfe et al., 2014). The present study focused solely on the manipulation of endurance training intensity during short-term concurrent training, while controlling for the influence of other potential confounding variables (e.g., divergences in endurance training volume, resistance and endurance training order, between-mode recovery, and endurance training modality). The possibility exists that endurance training intensity may play a greater or lesser role in mediating the interference effect if other concurrent training variables are differentially manipulated. Further work is required to elucidate the roles of these additional concurrent training variables in mediating the interference effect to inform further practical recommendations for mitigating the interference effect.

The potential for individual responses to concurrent training, and subsequently interference to RT adaptations, should also be considered in the context of the present data. It is clear from the variability in training-induced changes in performance measures (Figure 4) that there indeed appears to be responders and non-responders to the training intervention, supporting previous observations following both endurance (Bouchard and Rankinen, 2001) and RT (Hubal et al., 2005). It is recognized, however, that appropriate quantification of individual responses to controlled trials requires a large sample size or averaging of repeated measurements to compensate for a large error of measurement (Hopkins, 2015). Future studies should, where possible, incorporate study designs with larger sample sizes and repeated measurements of performance and morphological measures, which will subsequently improve the ability of the future studies to make clear inferences about individual responses to training.

## Conclusion

This is the first report of the effects of incorporating either HIT or work-matched MICT into a concurrent training program on adaptations of maximal strength, CMJ performance, aerobic capacity, and body composition compared with performing RT alone. In summary, it was demonstrated that HIT and MICT similarly attenuated the RT-induced increase in maximal lower-, but not upper-body, strength, as well as increases in peak CMJ force and power. These observations suggest that endurance training volume may be a more critical mediator of interference to maximal strength gain rather than training intensity, at least in moderately-trained individuals. Training-induced increases in lower-body lean mass were attenuated for the HIT+RT group relative to MICT+RT and RT, although the magnitude of between-group differences in lean mass gain were small. Total internal training load was higher for the HIT+RT group compared with the MICT+RT group, due primarily to a higher non-prescribed training load, which may have contributed to the attenuation of the lower-body lean mass gain for the HIT+RT group. Future work should further explore the role of endurance training volume in the interference effect, and whether low-volume HIT may confer benefits by minimizing interference when compared with higher volume HIT or MICT during periods of concurrent training.

## Key points

Little is known about the role of individual concurrent training variables in mediating interference between concurrent endurance and RT. We sought to clarify whether the intensity of endurance training was important in mediating interference to RT adaptations during short-term concurrent training. We show that interference to maximal strength gain is similar regardless of whether HIT or MICT cycling is incorporated into a concurrent training program, suggesting that on a work-matched basis, endurance training intensity is not a critical mediator of interference to maximal strength gain during short term concurrent training. The present data also lend support to the notion that endurance training volume may be a more important factor in mediating the interference effect during concurrent training.

## Author contributions

JF, JB, EH, DB, and NS designed the study. JF and EH performed data collection. JF, JB, EH, DB, and NS contributed to analysis and interpretation of data. JF, JB, EH, DB, and NS wrote and approved the final version of the manuscript.

### Conflict of interest statement

The authors declare that the research was conducted in the absence of any commercial or financial relationships that could be construed as a potential conflict of interest.
