# Supplementary material for: Endurance Training Intensity Does Not Mediate Interference to Maximal Lower-Body Strength Gain during Short-Term Concurrent Training
Source: Front Physiol. 2016 Nov 3;7:487. doi: 10.3389/fphys.2016.00487 (PMC5093324; doi:10.3389/fphys.2016.00487)
Supplement: Supplementary file 1 [file Table1.PDF]

Supplementary table 1. Summary of magnitude-based inference (MBI) data for all within-group comparisons.

Measure	Group	Mean PRE-POST change		Standardised effect size (ES)		Effect magnitude	Qualitative likelihood of true effect magnitude being substantial	*P* value
		% change	±90% CL	ES (*d*)	±90% CL			
**Maximal strength**								
1RM leg press	RT	38.5	8.5	1.26	0.24	large	most likely	< 0.001
	HIT+RT	28.7	5.3	1.17	0.19	moderate	most likely	< 0.001
	MICT+RT	27.5	4.6	0.81	0.12	moderate	most likely	0.001
1RM bench press	RT	20.5	6.2	0.50	0.14	small	most likely	< 0.001
	HIT+RT	15.9	2.6	0.62	0.09	moderate	most likely	< 0.001
	MICT+RT	14.8	2.3	0.39	0.06	small	most likely	< 0.001
**Counter-movement jump (CMJ) variables**								
Peak CMJ force	RT	7.4	3.4	0.46	0.20	small	very likely	0.008
	HIT+RT	0.1	3.6	0.00	0.23	trivial	unlikely	0.979
	MICT+RT	-0.8	4.9	-0.04	0.26	trivial	unlikely	0.790
Peak CMJ power	RT	12.6	10.5	1.09	0.85	moderate	very likely	0.035
	HIT+RT	3.2	5.6	0.20	0.34	small	possibly	0.266
	MICT+RT	5.0	6.1	0.19	0.23	trivial	possibly	0.241
Peak CMJ velocity	RT	9.6	8.2	0.29	0.24	small	likely	0.099
	HIT+RT	2.6	4.8	0.17	0.31	trivial	possibly	0.306
	MICT+RT	6.0	4.0	0.40	0.26	small	likely	0.015
Peak CMJ displacement	RT	9.5	10.0	0.22	0.22	small	possibly	0.108
	HIT+RT	7.8	9.1	0.50	0.56	small	likely	0.134
	MICT+RT	7.0	8.5	0.34	0.40	small	possibly	0.129

Measure	Group	Mean PRE-POST change		Standardised effect size (ES)		Effect magnitude	Qualitative likelihood of true effect magnitude being substantial	*P* value
		% change	±90% CL	ES (*d*)	±90% CL			
Maximal rate of force development	RT	25.4	26	0.43	0.39	small	likely	0.152
	HIT+RT	-4.9	11.8	-0.12	0.29	trivial	possibly	0.709
	MICT+RT	10	33.5	0.29	0.91	small	possibly	0.536
**Body composition**								
Lean mass (lower)	RT	4.1	2.0	0.33	0.16	small	likely	0.023
	HIT+RT	1.8	1.6	0.13	0.12	trivial	unlikely	0.069
	MICT+RT	3.6	2.4	0.45	0.30	small	likely	0.052
Lean mass (upper)	RT	0.4	1.9	0.02	0.12	trivial	very unlikely	0.719
	HIT+RT	1.4	2.0	0.13	0.17	trivial	unlikely	0.198
	MICT+RT	1.8	2.9	0.17	0.28	small	possibly	0.325
Lean mass (total)	RT	1.6	1.4	0.12	0.10	trivial	unlikely	0.102
	HIT+RT	1.6	1.1	0.14	0.09	trivial	unlikely	0.038
	MICT+RT	2.4	2.4	0.27	0.26	small	possibly	0.151
Body fat %	RT	-0.6	1.0	-0.08	0.17	trivial	unlikely	0.372
	HIT+RT	-0.2	0.9	-0.03	0.15	trivial	very unlikely	0.659
	MICT+RT	-0.9	1.0	-0.23	0.25	small	possibly	0.115

Measure	Group	Mean PRE-POST change		Standardised effect size (ES)		Effect magnitude	Qualitative likelihood of true effect magnitude being substantial	*P* value
		% change	±90% CL	ES (*d*)	±90% CL			
**Aerobic capacity**								
Absolute $\dot{V}O_{2peak}$	RT	-0.6	6.4	-0.02	0.21	trivial	unlikely	0.876
	HIT+RT	5.3	2.7	0.25	0.12	small	likely	0.162
	MICT+RT	6.1	5.0	0.27	0.22	small	possibly	0.103
Relative $\dot{V}O_{2peak}$	RT	-2.2	6.7	-0.06	0.17	trivial	unlikely	0.593
	HIT+RT	4.0	4.6	0.11	0.13	trivial	unlikely	0.320
	MICT+RT	5.0	5.4	0.18	0.18	trivial	possibly	0.131
Lactate threshold	RT	7.4	9.4	0.13	0.16	trivial	unlikely	0.161
	HIT+RT	8.3	6.5	0.20	0.15	small	possibly	0.054
	MICT+RT	12.6	8.0	0.30	0.18	small	likely	0.107
Peak aerobic power	RT	-2.2	6.5	-0.06	0.17	trivial	very unlikely	0.515
	HIT+RT	8.8	4.1	0.31	0.14	small	likely	0.010
	MICT+RT	4.9	4.8	0.19	0.18	trivial	possibly	0.096
