# Supplementary material for: Endurance Training Intensity Does Not Mediate Interference to Maximal Lower-Body Strength Gain during Short-Term Concurrent Training
Source: Front Physiol. 2016 Nov 3;7:487. doi: 10.3389/fphys.2016.00487 (PMC5093324; doi:10.3389/fphys.2016.00487)
Supplement: Supplementary file 2 [file Table2.PDF]

Supplementary table 2. Summary of magnitude-based inference (MBI) data for all between-group comparisons.

Measure	Group comparison	Mean difference in PRE-POST change		Standardised effect size (ES)		Effect magnitude	Qualitative likelihood of true effect magnitude being substantial
		% difference	±90% CL	ES (*d*)	±90% CL		
**Maximal strength**							
1RM leg press	HIT+RT vs. RT	-7.4	8.7	-0.40	0.40	small	likely
	MICT+RT vs. RT	-8.2	9.9	-0.60	0.45	moderate	likely
	HIT+RT vs. MICT+RT	0.9	8.1	0.03	0.30	trivial	unlikely
1RM bench press	HIT+RT vs. RT	-3.8	6.1	-0.04	0.22	trivial	possibly
	MICT+RT vs. RT	-4.7	6.1	-0.15	0.20	trivial	possibly
	HIT+RT vs. MICT+RT	-0.9	8.1	-0.03	0.19	trivial	unlikely
**Counter-movement jump (CMJ) variables**							
Peak CMJ force	HIT+RT vs. RT	-6.8	4.5	-0.41	0.28	small	likely
	MICT+RT vs. RT	-9.9	11.2	-0.54	0.65	small	likely
	HIT+RT vs. MICT+RT	-5.0	12.1	-0.33	0.19	small	possibly
Peak CMJ power	HIT+RT vs. RT	-5.1	7.3	-0.38	0.56	small	possibly
	MICT+RT vs. RT	-3.5	8.7	-0.21	0.54	small	possibly
	HIT+RT vs. MICT+RT	1.7	8.4	0.10	0.48	trivial	possibly
Peak CMJ velocity	HIT+RT vs. RT	-6.4	9.1	-0.32	0.46	small	possibly
	MICT+RT vs. RT	-3.3	9.3	-0.16	0.46	trivial	possibly
	HIT+RT vs. MICT+RT	1.4	5.6	0.15	0.61	trivial	possibly

Measure	Group comparison	Mean difference in PRE-POST change		Standardised effect size (ES)		Effect magnitude	Qualitative likelihood of true effect magnitude being substantial
		% difference	±90% CL	ES (*d*)	±90% CL		
Peak CMJ displacement	HIT+RT vs. RT	-1.6	11.6	-0.06	0.43	trivial	possibly
	MICT+RT vs. RT	-7.3	10.4	-0.28	0.41	small	possibly
	HIT+RT vs. MICT+RT	5.8	9.8	0.42	0.74	small	possibly
Maximal rate of force development	HIT+RT vs. RT	24.1	26.1	0.72	0.88	moderate	likely
	MICT+RT vs. RT	12.3	32.4	0.24	0.66	small	possibly
	HIT+RT vs. MICT+RT	0.8	57.1	0.02	1.38	trivial	possibly
**Body composition**							
Lean mass (lower)	HIT+RT vs. RT	-2.2	2.8	-0.18	0.23	trivial	possibly
	MICT+RT vs. RT	-0.5	3.5	-0.05	0.35	trivial	unlikely
	HIT+RT vs. MICT+RT	1.7	3.1	0.16	0.28	trivial	possibly
Lean mass (upper)	HIT+RT vs. RT	1.0	2.5	0.08	0.19	trivial	unlikely
	MICT+RT vs. RT	1.4	3.5	0.10	0.26	trivial	possibly
	HIT+RT vs. MICT+RT	0.4	3.5	0.04	0.34	trivial	unlikely
Lean mass (total)	HIT+RT vs. RT	0.1	1.9	0.01	0.15	trivial	very unlikely
	MICT+RT vs. RT	0.8	3.1	0.06	0.27	trivial	unlikely
	HIT+RT vs. MICT+RT	0.8	2.9	0.08	0.29	trivial	unlikely
Body fat %	HIT+RT vs. RT	2.0	8.0	0.05	0.20	trivial	unlikely
	MICT+RT vs. RT	1.9	7.8	0.06	0.24	trivial	unlikely
	HIT+RT vs. MICT+RT	3.8	6.8	0.12	0.23	trivial	possibly

Measure	Group comparison	Mean difference in PRE-POST change		Standardised effect size (ES)		Effect magnitude	Qualitative likelihood of true effect magnitude being substantial
		% difference	±90% CL	ES (*d*)	±90% CL		
**Aerobic capacity**							
Absolute $\dot{V}O_{2peak}$	HIT+RT vs. RT	5.9	9.4	0.26	0.40	small	possibly
	MICT+RT vs. RT	6.8	9.3	0.27	0.35	small	possibly
	HIT+RT vs. MICT+RT	0.8	8.1	0.04	0.38	trivial	unlikely
Relative $\dot{V}O_{2peak}$	HIT+RT vs. RT	6.3	10.2	0.19	0.30	trivial	possibly
	MICT+RT vs. RT	7.4	9.4	0.24	0.29	small	possibly
	HIT+RT vs. MICT+RT	1.0	8.3	0.04	0.29	trivial	unlikely
Lactate threshold	HIT+RT vs. RT	0.9	10.2	0.02	0.24	trivial	unlikely
	MICT+RT vs. RT	4.9	14.6	0.12	0.35	trivial	possibly
	HIT+RT vs. MICT+RT	4.0	13.7	0.11	0.37	trivial	possibly
Peak aerobic power	HIT+RT vs. RT	11.3	8.1	0.35	0.24	small	likely
	MICT+RT vs. RT	7.3	7.8	0.24	0.25	small	possibly
	HIT+RT vs. MICT+RT	-3.6	5.9	-0.16	0.26	trivial	possibly
